# Patterns of airway inflammation and MMP-12 expression in smokers and ex-smokers with COPD

**DOI:** 10.1186/1465-9921-8-81

**Published:** 2007-11-14

**Authors:** Agne Babusyte, Kristina Stravinskaite, Jolanta Jeroch, Jan Lötvall, Raimundas Sakalauskas, Brigita Sitkauskiene

**Affiliations:** 1 Laboratory of Pulmonology, Institute for Biomedical Research, Kaunas University of Medicine, Eiveniu 4, LT-50009, Kaunas, Lithuania; 2 Department of Pulmonology and Immunology, Kaunas University of Medicine, Eiveniu 2, LT-50009, Kaunas, Lithuania; 3 The Lung Pharmacology Group, Department of Respiratory Medicine and Allergology, Institute of Internal Medicine, Göteborg University, Guldhedsgatan 10A, 413 46 Gothenburg, Sweden

## Abstract

**Background:**

Smoking activates and recruits inflammatory cells and proteases to the airways. Matrix metalloproteinase (MMP)-12 may be a key mediator in smoke induced emphysema. However, the influence of smoking and its cessation on airway inflammation and MMP-12 expression during COPD is still unknown. We aimed to analyse airway inflammatory cell patterns in induced sputum (IS) and bronchoalveolar lavage (BAL) from COPD patients who are active smokers and who have ceased smoking >2 years ago.

**Methods:**

39 COPD outpatients – smokers (n = 22) and ex-smokers (n = 17) were studied. 8 'healthy' smokers and 11 healthy never-smokers were tested as the control groups. IS and BAL samples were obtained for differential and MMP-12^+^-macrophages count analysis.

**Results:**

The number of IS neutrophils was higher in both COPD groups compared to both controls. The amount of BAL neutrophils was higher in COPD smokers compared to healthy never-smokers. The number of BAL MMP-12^+^-macrophages was higher in COPD smokers (1.6 ± 0.3 × 10^6^/ml) compared to COPD ex-smokers, 'healthy' smokers and healthy never-smokers (0.9 ± 0.4, 0.4 ± 0.2, 0.2 ± 0.1 × 10^6^/ml respectively, p < 0.05).

**Conclusion:**

The lower amount of BAL neutrophils in COPD ex-smokers, compared to COPD smokers, suggests positive alterations in alveolar compartment after smoking cessation. Smoking and disease itself may stimulate MMP-12 expression in airway compartments (IS and BAL) from COPD patients.

## Background

Smoking is the major known risk factor for the development of chronic obstructive pulmonary disease (COPD), which is characterized by progressive and not fully reversible airflow limitation [1]. The pathogenesis of COPD is multifactor, involving airway inflammation, associated with an infiltration of inflammatory cells and protease-antiprotease imbalance [2,3].

Over 85% COPD patients have been regular smokers [4,5]. It is well known, that inflammation initiated by smoking leads to a changes in both – airways and lung parenchyma. The main known contribution of smoking is activation and recruitment of inflammatory cells to the lungs [6-8]. We have previously observed a tendency of neutrophils to be increased in the airways of stable COPD patients [9]. Other studies have also shown that cigarette smoke produces an increase of neutrophils in bronchoalveolar lavage (BAL) and lung tissue [10-12]. Although, the major environmental risk factor – smoking, for COPD development is well known, the changes of COPD induced by inflammation after smoking cessation are less evaluated.

It was also suggested, that various metalloproteinases (MMPs), especially MMP-2 and MMP-9, mediate airway inflammation and remodelling [13-15]. Since, it is nearly impossible to investigate which individual MMP is the most important in COPD pathogenesis. MMP-12 was first detected as an elastolytic proteinase in alveolar macrophages of cigarette smokers [16]. Whilst, animal studies have shown that MMP-12 is important in cigarette smoke induced emphysema [17-19], the relevance of MMP-12 in human disease is controversial.

Thus, we aimed to analyse airway inflammatory cell patterns in smokers and ex-smokers with COPD and to compare whether it differs from 'healthy' smokers and never-smokers. Also, according to a previous study, showing an increase in MMP-12 in the induced sputum (IS) of COPD patients [20], we have assessed an expression of MMP-12 in IS and BAL cells from these COPD and healthy subjects groups. Furthermore, we analysed if the decline of pulmonary function in COPD patients is related to the smoking history and MMP-12 expression in airway cells.

## Methods

### Study population

We studied 39 outpatients with stable COPD, according to GOLD (stage II-III) [1]. All patients met following criteria: has not used inhaled and systemic steroids at least 1 month before the study and had more than 20 pack-years smoking history. None of the subjects showed signs of acute respiratory infection at least one month before the investigation. All patients were screened for deficiency of alfa-1 antitrypsin (AAT) by quantitative ELISA test (Eurodiagnosta, Sweden) and was established, that none of the patients had the Z allele, which may cause the deficiency of AAT. The patients were divided into 2 groups: COPD smokers (n = 22), who are currently smokers and COPD ex-smokers (n = 17), who ceased smoking at least 2 years before investigation (however, we did not test a cotinine level to ensure, if they have really ceased smoking).

8 smokers without airways obstruction ('healthy' smokers) and 11 healthy never-smokers with normal lung function were tested as control groups.

Smoking history was calculated in pack-years as the product of tobacco use (in years) and the average number of cigarettes smoked per day/20 (years × cig. per day/20).

The study was approved by the Regional Bioethics Committee in Kaunas University of Medicine and written informed consent was received from all participants.

### Lung function testing

Pulmonary function was tested using a pneumotachometric spirometer "CustovitM" (Custo Med, Germany) with subjects in the sitting position, and the highest value of forced expiratory volume in 1 sec (FEV_1_) and forced vital capacity (FVC) from at least two technically satisfactory maneuvers differing by less than 5% was recorded. Normal values were characterized according to Quanjer and colleagues [21]. Subjects had to avoid the use of short-acting β_2_-agonists at least 8 h prior the test.

### Sputum induction and processing

After lung function test, subjects inhaled 10 mL of sterile hypertonic saline solution (3%, 4% or 5% NaCl (Ivex Pharmaceuticals, USA)) at room temperature (RT) from an ultrasonic nebulizer (DeVilbiss Health Care, USA). The duration of each inhalation was 5 min and was stopped after expectoration an adequate amount of sputum. Spirometry was performed after each inhalation, in order to detect a possible decrease of FEV_1_. Sputum was poured into a Petri dish and separated from saliva. A fourfold volume of freshly prepared 0.1% dithiothreitol (DTT; Sigma-Aldrich, Germany) was added. The mixture was vortexed and placed on a bench rocker for 15 min. at RT. Next, an equal volume of phosphate-buffered saline (PBS; Sigma-Aldrich, Germany), solution was added to the DTT. The cell pellet was separated using 40 μm cell stainer (Becton Dickinson, USA). The mixture was centrifuged for 10 min at 4°C, the supernatant was aspirated and stored at -70°C for later assay.

The total cell counts, percentage of epithelial cells and cell viability were investigated using a Neubauer hemocytometer (Heinz-Herenz; Germany) by microscope (B5 Professional, Motic, China), using Trypan blue exclusion method. Cytospin samples of induced sputum were prepared using a cytofuge instrument (Shandon Southern Instruments, USA). The cytospin preparations for immunocytochemistry were air dried for 2 h and stored at -70°C until further investigation.

### Bronchoscopy and BAL processing

Bronchoscopy was performed in a week after sputum induction procedure. Subjects were not allowed to drink or eat at least 4 h, to smoke at least 10 h before the procedure. To perform BAL, the local upper airways anesthesia with 5 mL of 2% lidocaine (Grindex, Latvia) was used. All bronchoscopic examinations were performed in the morning. The bronchoscope (Olympus, USA) was wedged into the segmental bronchus of the middle lobe and 20 mL × 7, a total 140 mL of sterile saline solution (0.9% NaCl) was infused. Fluid was gently aspirated immediately after the infusion has been completed and was collected into a sterile container. The fluid was immediately filtered using 40 μm cell stainer (Becton Dickinson, USA) and centrifuged at 4°C for 10 min. Supernatants were removed and frozen at -70°C for further investigation. Preparation of BAL cytospins was the same as the preparation of IS samples described above.

### Cell analysis

Prepared IS and BAL cytospins were stained by the May-Grünwald-Giemsa method for differential cell counts. Cell differentiation was determined by counting approximately 400 cells in random fields of view under light microscope, excluding squamous epithelial cells. The cells were identified using standard morphological criteria, by nuclear morphology and cytoplasmic granulation. Cell counts were expressed as percentages of total cells and absolute values (10^6^/ml).

### MMP-12 immunocytochemistry (ICC)

**Figure 1:**
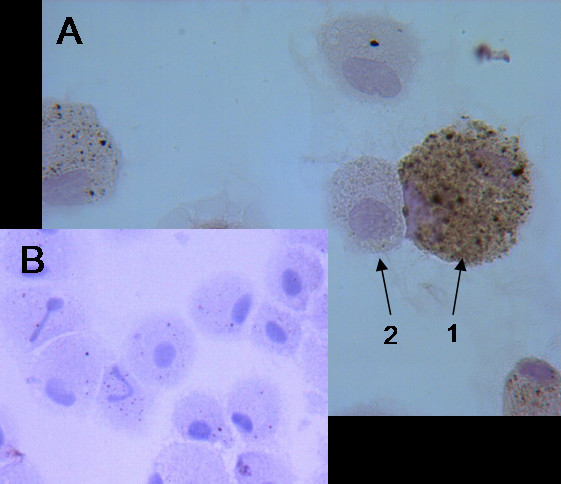
**MMP-12 expression in BAL**. Representative photomicrograph (original magnification: ×1000) of BAL cells immunocytochemical staining for MMP-12 (brown cytoplasm). 1 – MMP-12^+^-macrophage, 2 – MMP-12^-^-macrophage. A – positive control, B – negative control (rabbit IgG).

MMP-12 expression in IS and BAL cytospin preparations was detected immunocytochemically. Cytospin preparations were fixed in 4% paraphormaldehyde (Merck, USA) in PBS for 20 min. and subsequently washed in PBS. All incubations were performed at RT. Non-specific binding sites were blocked with 5% normal blocking serum (Goat ABC Staining System, Santa Cruz, USA) for 35 min. The slides were incubated with optimum concentration of goat anti-human MMP-12 antibody (Santa Cruz, USA), which is raised against a peptide mapping near the C-terminus of MMP-12, and negative control (rabbit IgG, Santa Cruz, USA) for 30 min. After washings in PBS, the slides were incubated with biotinylated secondary antibody (Santa Cruz, USA) for 30 min. Followed by washings in PBS, slides were incubated with avidin-biotinylated peroxydase (Santa Cruz, USA) complex for 35 min. After washings, the staining with chromogenic substrate 3,3'diaminobenzidine system (Santa Cruz, USA) was developed for 10–15 min monitoring under light microscope. The slides were counterstained with Mayer's haematoxylin (Sigma-Aldrich, Germany) for 1–2 min and mounted in Crystal Mounting Medium (Santa Cruz, USA). All slides were evaluated under light microscope in random fields of view counting up to 300–400 cells. Morphologically, all MMP-12 expressing cells were macrophages. Macrophages with brown staining in cytoplasm were counted as MMP-12 positive macrophages (MMP-12^+^-macrophages) (Fig. 1A). Figure 1B represents the negative staining with rabbit IgG. The absolute amount of MMP-12^+^-macrophages (10^6^/ml) was calculated according to the number of MMP-12^+^-macrophages and total inflammatory cell count. The intensity of staining was evaluated as: 0 – negative; +++ – very strong expression. The variations MMP-12^+^-macrophages were counted by two "blinded" researchers and the mean of their results was calculated. In most cases, the variation of cell count between examinators was less than 5%.

It is important to note, that we used DTT for preparation of IS samples, which may interfere with expression of MMP-12. Therefore, we have compared a preparation of few IS samples for MMP-12 and inflammatory cell count with DTT and without it, and we did not notice any significant differences.

### Statistical analysis

Statistical analysis was performed using Statistical Package for the Social Sciences, version 12.0 for Windows (SPSS 12.0). Data was expressed as the mean of percentage or absolute value (10^6^/ml) ± standard error of mean (SEM). Differences between all groups were explored using one-way ANOVA followed by Kruskal-Wallis test. Mann-Whitney U-test was used to assess the statistical significance of differences between the groups. A P-value < 0.05 was considered significant. Correlations between analysed parameters were assessed using Spearman's rank coefficient.

## Results

### Characteristics of subjects

The average age did not differ between investigated groups (Table 1). The number of pack-years did not significantly differ between COPD smokers, COPD ex-smokers and 'healthy' smokers. Lung function parameters did not differ between COPD groups, but were lower compared to controls.

**Table 1:** Characteristics of subjects

**Variables**	**COPD smokers**	**COPD ex-smokers**	**'Healthy' smokers**	**Healthy never-smokers**
Subjects (n)	22	17	8	11
Male/Female	22/0	14/3	7/1	4/7
Age (years)	64.2 ± 4.9	62.7 ± 6.3	61.7 ± 6.2	59.8 ± 8.2
Smoking (pack-years)	33.4 ± 5.7	27.9 ± 5.1	28.8 ± 12.1	-
FEV_1 _(L)	1.5 ± 0.4*^#^	1.7 ± 0.2*^#^	3.2 ± 0.6	3.7 ± 0,2
FEV_1 _(% pred.)	53.3 ± 4.2*^#^	57.1 ± 4.7*^#^	109.6 ± 5.3	117.5 ± 4.1
FVC (L)	2.7 ± 0.5*^#^	2.9 ± 0.4*^#^	3.0 ± 0.2	3.3 ± 0.2
FVC (% pred.)	69.8 ± 9.1*^#^	71.7 ± 7.3*^#^	108.1 ± 8.2	110.0 ± 6.4
FEV_1_/FVC ratio	50.2 ± 5.9*^#^	52.5 ± 6.8*^#^	91.0 ± 4.6	93.5 ± 1.0

Values are mean of percentage ± SEM. *: p < 0.05 compared to healthy never-smokers; ^#^: p < 0.05 compared to 'healthy' smokers

### Cellular composition of IS

**Figure 2:**
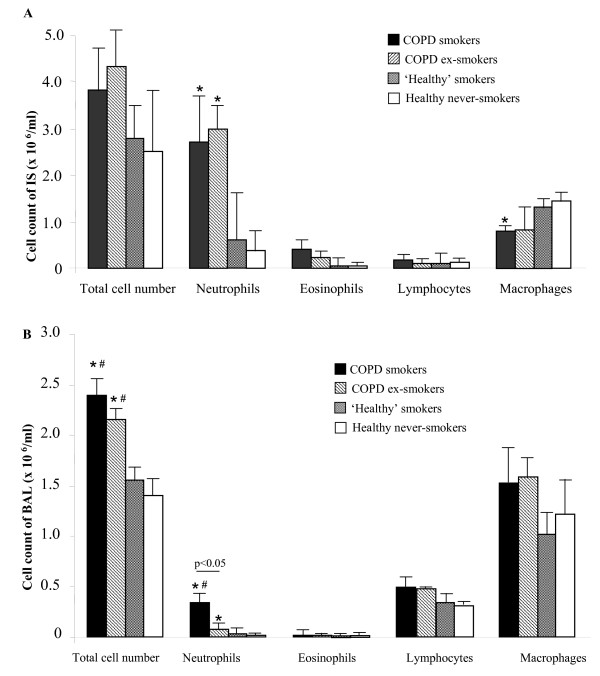
**Differential cell counts in IS and BAL (10^6^/ml)**. Differential cell composition in IS (A) and BAL (B) from COPD smokers, COPD ex-smokers, 'healthy' smokers and healthy never-smokers. Data are shown as mean ± SEM. *p < 0.05 compared to healthy never-smokers, #p < 0.05 compared to 'healthy' smokers.

The total cell count of IS did not differ between all groups (Fig. 2A). The composition of inflammatory cells did not differ between COPD smokers and COPD ex-smokers. COPD groups showed a predominance of neutrophils, compared to both healthy subjects groups in percentages (Table 2). An absolute amount of these cells was higher in COPD smokers and COPD ex-smokers compared to healthy never-smokers, but not 'healthy' smokers (Fig. 2A).

**Table 2:** Differential cell counts in IS and BAL samples

**Variable**	**COPD smokers**	**COPD ex-smokers**	**'Healthy' smokers**	**Healthy never-smokers**	**CS/CE**	**CS/HS**	**CS/HN**	**CE/HS**	**CE/HN**	**HS/HN**
**Induced sputum**										

**Neutrophils**	67.7 ± 7.7	75.9 ± 9.5	22.6 ± 3.3	16.1 ± 7.0	>0.05	**<0.05**	**<0.01**	**0.05**	**0.01**	>0.05
**Eosinophils**	4.5 ± 2.2	3.4 ± 1.6	1.8 ± 0.4	2.3 ± 0.5	>0.05	>0.05	>0.05	>0.05	>0.05	>0.05
**Lymphocytes **	4.7 ± 1.6	2.7 ± 0.9	6.3 ± 1.1	4.9 ± 0.8	>0.05	>0.05	>0.05	>0.05	>0.05	>0.05
**Macrophages **	23.1 ± 7.5	18.0 ± 3.5	69.3 ± 9.5	64.8 ± 10.2	>0.05	**<0.05**	**<0.05**	**0.05**	**<0.05**	>0.05

**BAL**										

**Recovery of BAL fluid **	43.1 ± 8.3	59.3 ± 6.7	83.3 ± 6.9	81.0 ± 3.8	**<0.05**	**<0.05**	**<0.05**	**<0.05**	**<0.05**	**>0.05**
**Neutrophils**	17.4 ± 4.8	3.2 ± 1.5	2.7 ± 0.4	1.2 ± 0.5	**<0.01**	**0.05**	**<0.01**	>0.05	**0.01**	**<0.01**
**Eosinophils **	0.8 ± 0.3	0.8 ± 0.3	0.2 ± 0.1	0.2 ± 0.1	>0.05	>0.05	>0.05	>0.05	**<0.05**	>0.05
**Lymphocytes **	22.6 ± 6.5	19.8 ± 3.9	24.2 ± 4.5	21.0 ± 4.4	>0.05	>0.05	>0.05	>0.05	>0.05	>0.05
**Macrophages**	59.2 ± 6.9	76.2 ± 9.3	72.9 ± 10.6	77.6 ± 10.6	**<0.01**	>0.05	**<0.05**	>0.05	>0.05	>0.05

Values are mean percentage of total cells ± SEM. CS: COPD smokers; CE: COPD ex-smokers; HS: 'healthy' smokers; HN: healthy never-smokers.

Macrophages in IS were more obvious in 'healthy' smokers and healthy never-smokers, due to higher percentage of neutrophils in both COPD groups. The percentage of macrophages was significantly lower in COPD groups compared to both healthy subjects groups, and did not significantly differ between both COPD and between both controls groups. An absolute amount of macrophages in COPD smokers was lower compared to healthy never-smokers and did not significantly differ from 'healthy' smokers, however a tendency was seen (p = 0.06) (Fig. 2A).

### Cellular composition of BAL

The total BAL cell number was higher in COPD groups, compared to healthy subjects groups, while it did not differ between COPD smokers and COPD ex-smokers and between 'healthy' smokers and healthy never-smokers (Fig. 2B). Also, the recovery of BAL was significantly higher in COPD ex-smokers, compared to COPD smokers (Table 2). While, this volume was significantly higher in both healthy subjects groups, than in COPD smokers and COPD ex-smokers. The recovery of BAL did not differ between both 'healthy' smokers and healthy never-smokers.

The percentage of neutrophils was increased in COPD smokers, compared to COPD ex-smokers and healthy subjects groups. Whereas, the percentage of these inflammatory cells in COPD ex-smokers was higher compared to healthy never-smokers, but did not differ from 'healthy' smokers. The percentage of BAL neutrophils in 'healthy' smokers was also higher than in healthy never-smokers. The absolute amount of neutrophils in COPD smokers was higher compared to all other groups (Fig. 2B).

### Expression of MMP-12 in IS and BAL cells

**Figure 3:**
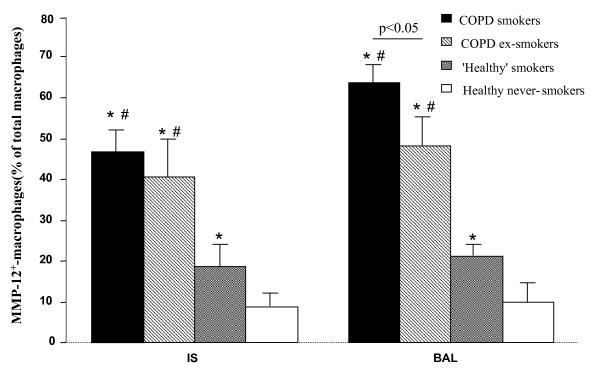
**MMP-12^+^-macrophages in IS and BAL**. The relative number of MMP-12^+^-macrophages in IS and BAL samples from COPD smokers, COPD ex-smokers, 'healthy' smokers and healthy never-smokers. Data are shown as mean ± SEM. *p < 0.05 compared to healthy never-smokers, #p < 0.05 compared to 'healthy' smokers.

An immunocytochemical staining of IS cells for MMP-12 did not show significant differences between COPD smokers and COPD ex-smokers neither in percentages (Fig. 3), nor in absolute values. The percentage of IS MMP-12^+^-macrophages was higher in COPD groups compared to healthy subjects groups. 'Healthy' smokers had higher percentage of these cells than healthy never-smokers (Fig. 3), but the absolute amount of MMP-12^+^-macrophages did not differ.

The amount of BAL MMP-12^+^-macrophages was also significantly higher in COPD groups than in controls in percentages and absolute values. Furthermore, the number of BAL MMP-12^+^-macrophages was higher in COPD smokers compared to COPD ex-smokers, and in 'healthy' smokers compared to healthy never-smokers (Fig. 3).

Analysing the BAL samples we have observed macrophages differentiating in size and granularity of cytoplasm, while we did not evaluate the relations of MMP-12 expression with their morphology.

### Smoking history relation with cellular patterns, MMP-12 expression and lung function parameters

**Figure 4:**
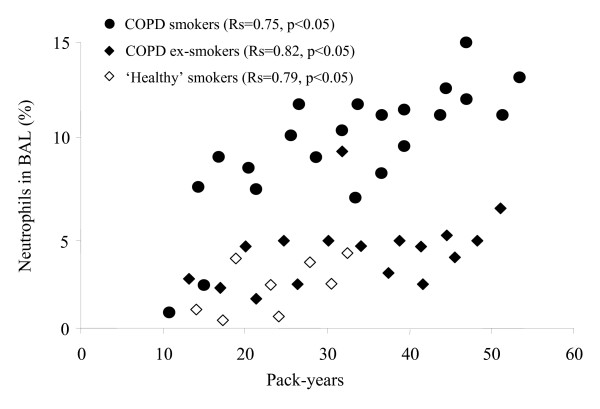
**Smoking history and neutrophils**. Correlation between smoking history (pack-years) and neutrophils (%) in BAL samples from COPD smokers, COPD ex-smokers and 'healthy' smokers.

The number of pack-years correlated with FEV_1 _(%) in COPD smokers (R = -0.70, p < 0.05) and 'healthy' smokers (R = -0.61, p < 0.05). Also, the pack-years correlated with IS neutrophils in COPD ex-smokers (R = 0.66, p < 0.05). A correlation between pack-years and BAL neutrophils in COPD smokers, COPD ex-smokers and 'healthy' smokers groups (Fig. 4) was also obtained. Moreover, the pack-years correlated with BAL macrophages in COPD smokers (R = 0.87, p < 0.05) and 'healthy' smokers (R = 0.68, p < 0.05). These parameters did not correlate with IS inflammatory cells.

The number of IS macrophages negatively correlated with FEV_1 _(%) in COPD smokers (R = -0.53, p < 0.05) and COPD ex-smokers (R = -0.58, p < 0.05). The correlation between BAL macrophages and FEV_1 _(%) in all studied groups was also obtained (R = -0.88; -0.62; -0.67; -0.78, p < 0.05 respectively).

**Figure 5:**
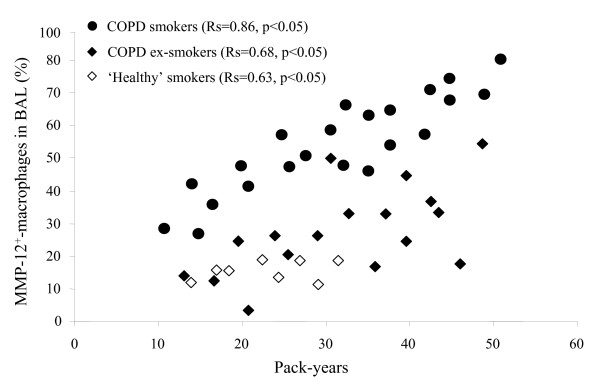
**Smoking history and MMP-12^+^-macrophages**. Correlation between smoking history (pack-years) and MMP-12^+^-macrophages (%) in BAL samples from COPD smokers, COPD ex-smokers and 'healthy' smokers (p < 0.05).

The number of pack-years correlated with IS MMP-12^+^-macrophages in COPD smokers (R = 0.54, p < 0.05), COPD ex-smokers (R = 0.64, p < 0.05) and 'healthy' smokers (R = 0.78, p < 0.05). Much stronger correlation between pack-years and BAL MMP-12^+^-macrophages was obtained (Fig. 5).

## Discussion

We aimed to analyse the patterns of airway inflammation in COPD patients depending on their smoking status, and compare it to smokers without airways obstruction ('healthy' smokers) and healthy never-smokers. We have evaluated different tissue compartments (IS and BAL), as IS is thought to be a combination of resident mucus [22] and the composition of its cells may be influenced by inflammation in proximal airways [22]. While BAL cellular composition represents mainly the alveolar compartment [23-25], however this method usually is limited due invasiveness. We have analysed IS sputum and BAL, because differences in these patterns are still unclear. Also, we have analysed whether the possible differences in MMP-12 expression are influenced by smoking history and its cessation, as previous animal [17-19] and human [26,27] studies showed, that smoking exposure may increase an expression of MMP-12.

The number and composition of IS inflammatory cells did not significantly differ between smokers and ex-smokers with COPD, while the number of neutrophils was increased compared to healthy subjects. These results are in agreement with major previous studies [23,28], which have shown that cellular inflammatory response in COPD is characterized by an increase of total inflammatory cells, especially neutrophils, macrophages and lymphocytes in small and large airways [2,3]. Thus, our results may indicate the similar inflammatory response in smokers and ex-smokers with COPD, which is associated not only with smoking, but also with systemic inflammation. Influence of smoking may explain an increased number of BAL neutrophils in COPD smokers, compared to COPD ex-smokers, 'healthy' smokers and healthy never-smokers. Interestingly, the number of BAL neutrophils did not differ between COPD ex-smokers and 'healthy' smokers, while the amount of these cells was increased compared to healthy never-smokers. This finding supports the hypothesis, that cigarette smoking may cause cellular alterations [3,22], which may intensify an inflammation process, induced by disease itself.

Macrophage is predominant cell in IS from healthy never-smokers and 'healthy' smokers as well. The lower relative number of these cells obtained in COPD groups may indicate an ongoing inflammatory process. Also, the similar amount of BAL macrophages in COPD ex-smokers, 'healthy' smokers and never-smokers, suggests the possibility of positive alterations in the alveolar compartment after smoking cessation.

Also, we have obtained a higher recovery of BAL fluid in healthy subjects, compared to both COPD groups. According to Lofdahl *et al*. [29] suggestions, the extent of emphysema (measured as an emphysema index and the carbon monoxide diffusing capacity of the lung) may predict a low BAL recovery in patients with moderate-to-severe COPD. Moreover, the lower recovery of BAL fluid in COPD smokers than in COPD ex-smokers may indicate an increased inflammatory process in alveolar compartment strengthened by smoking. Furthermore, differences in BAL cell composition between COPD smokers and ex-smokers encouraged us to evaluate a correlation between smoking history, pulmonary function and inflammatory cells. We obtained, that smoking history (pack-years) positively correlates with number of BAL neutrophils in both COPD groups and 'healthy' smokers. Such relation once more supports the role of neutrophils recruitment in response to cigarette smoke and suggests that longer smoking history leads to more serious lung function damage. Smoking may have accumulative effect of inflammatory cells and may increase an inflammatory response in COPD and 'healthy' smokers as well. Also, we observed the positive correlation between BAL macrophages and smoking history in COPD smokers and 'healthy' smokers. It is known that cigarette smoke increases protease-antiprotease imbalance and alveolar macrophages, which are significant source of some MMPs [16,18]. According to animal studies, MMP-12 deficiency protects against cigarette smoke induced emphysema [18,19]. Though, most studies investigating MMP-12 were performed using animal models and exact role of MMP-12 in human COPD inflammation is not fully understood.

We analysed an expression of MMP-12 active form using immunocytochemistry.

The number of IS MMP-12^+^-macrophages did not differ between COPD groups, but it was higher compared to healthy subjects. Absence of significant differences in MMP-12 expression in IS may be explained by predominance of neutrophils, in COPD smokers and ex-smokers, which obviously do not express MMP-12. An expression of MMP-12 in IS from 'healthy' smokers was increased, compared to never-smokers, supporting the suggestion that smoking may increase an expression of this enzyme. Our results are in agreement to Demedts *et al*. [20], who found an increased sputum MMP-12 level in COPD patients, compared to healthy smokers, former smokers (>1 year) and never smokers, while they have not divided COPD patients into smokers and ex-smokers. Also, Molet et al., have reported an increase of MMP-12 in BAL and bronchial biopsies of COPD patients compared to controls [30], while they have not investigated an expression of MMP-12 according to smoking status.

One of the most interesting our findings was an increased number of MMP-12^+^-macrophages in BAL from COPD smokers compared to COPD ex-smokers. Also, the number of MMP-12^+^-macrophages was increased in both COPD groups, compared to controls. Nevertheless we observed a lower amount of BAL macrophages in COPD smokers, compared to COPD ex-smokers, the absolute and relative number of BAL MMP-12^+^-macrophages in COPD smokers was higher than in COPD ex-smokers. 'Healthy' smokers had higher number of BAL MMP-12^+^-macrophages, than never-smokers supporting the fact of smoking impact in MMP-12 expression. Actually, we did not evaluate the activity of macrophages in this study, thus we were not able to investigate the ratio of MMP-12 release and activated macrophages in this study.

Also, an increased number of BAL MMP-12^+^-macrophages in COPD ex-smokers, compared to 'healthy' smoking subjects, let us hypothesize that MMP-12 expression is induced not only by cigarette smoking, but may be an obligatory to the development of COPD.

Previous studies have shown that contribution of MMP-12 to smoke induced emphysema is probably enhanced by indirect effects, such as inactivation of AAT [31] and MMP-12 mediated recruitment of neutrophils to the lung [18]. Otherwise, our data suggests that MMP-12 may accumulate and do not rapidly decreases or inactivates after smoking cessation, exaggerating a persistent inflammation. An increased expression of MMP-12 in 'healthy' smokers, also may be a reason for COPD development in the future.

## Conclusion

Smokers and ex-smokers with COPD had close to similar number and type of IS inflammatory cells, indicating an ongoing inflammation in proximal airways after smoking cessation. Although, the lower amount of BAL neutrophils in COPD ex-smokers, compared to COPD smokers suggests, that smoking cessation may cause positive alterations in alveolar compartment.

Also, a higher number of MMP-12^+^-macrophages in IS and BAL from COPD smokers and COPD ex-smokers, indicates that smoking, which is an initial step contributing to the development of COPD, may stimulate MMP-12 expression in airway cells. Moreover, it let as argue that MMP-12 expression may be induced not only by smoking, but by the disease itself. A lower amount of BAL MMP-12^+^-macrophages and other mentioned inflammatory cells, compared to COPD smokers, may indicate a decrease of alveolar inflammation after smoking cessation.

## Abbreviations

BAL bronchoalveolar lavage

COPD chronic obstructive pulmonary disease

DTT dithiothreitol

FEV_1 _forced expiratory volume in 1 sec.

FVC forced vital capacity

ICC immunocytochemistry

IS induced sputum

MMP-12 matrix metalloproteinase

PBS phosphate-buffered saline

RT room temperature

## Competing interests

The author(s) declare that they have no competing interests.

## Authors' contributions

AB carried out the major part of cytological analysis and immunocytochemistry, participated in the writing of manuscript;

KS carried out screening and clinical evaluation of study subjects;

JJ participated in the study design, carried out the part of immunocytochemistry and performed some statistical analysis;

JL participated in the study design and in the sequence alignment

RS participated in the study design and in the sequence alignment

BS conceived and supervised the study and participated in its design, participated in the writing of the manuscript.

All authors read and approved the final manuscript.
